# Valorization of Wheat Bran by Co-Cultivation of Fungi with Integrated Hydrolysis to Provide Sugars and Animal Feed

**DOI:** 10.3390/biotech13020015

**Published:** 2024-05-18

**Authors:** Fabian Mittermeier, Fabienne Fischer, Sebastian Hauke, Peter Hirschmann, Dirk Weuster-Botz

**Affiliations:** 1 Chair of Biochemical Engineering, Technical University of Munich, 85748 Garching, Germany; 2 Bavarian Milling Confederation (Bayerischer Müllerbund e.V.), 80333 Munich, Germany

**Keywords:** co-cultivation, fungal processes, hydrolysis, wheat bran, waste-free bioprocesses

## Abstract

The enzymatic hydrolysis of agricultural residues like wheat bran enables the valorization of otherwise unused carbon sources for biotechnological processes. The co-culture of *Aspergillus niger* and *Trichoderma reesei* with wheat bran particles as substrate produces an enzyme set consisting of xylanases, amylases, and cellulases that is suitable to degrade lignocellulosic biomass to sugar monomers (D-glucose, D-xylose, and L-arabinose). An integrated one-pot process for enzyme production followed by hydrolysis in stirred tank bioreactors resulted in hydrolysates with overall sugar concentrations of 32.3 g L^−1^ and 24.4 g L^−1^ at a 25 L and a 1000 L scale, respectively, within 86 h. Furthermore, the residual solid biomass consisting of fermented wheat bran with protein-rich fungal mycelium displays improved nutritional properties for usage as animal feed due to its increased content of sugars, protein, and fat.

## 1. Introduction

For a few decades now, modern industries have had to face a variety of changes and challenges such as climate change, shortage of fossil raw materials, or the overproduction of waste [1,2]. Thus, one of the great goals is the replacement of conventional, fossil-based processes by sustainable bioprocesses based on renewable resources [3]. In order to further evade competition between the production of chemicals and food resources, in analogy to the so-called food-versus-fuel debate, the conversion and valorization of agricultural residues can pose another step forward. Additionally, the utilization of these materials supports the aim of circular and waste-free economies [4,5,6].

One of the most abundant agricultural residues is the outer layers of wheat grains, the wheat bran, with an annual world production of around 150 million tons [7]. Wheat bran originates from flour production and is mostly burned or used as an animal feed additive [8,9]. Its composition of the polysaccharides hemicellulose, cellulose, and starch, as well as proteins, poses a nearly unused reservoir of carbohydrates usable as substrates in bioprocesses. However, the enzymatic degradation of those structures into easily convertible sugars is complex and requires different enzymes with a broad spectrum of hydrolytic activities.

The hemi-cellulosic compound mostly present in wheat bran is arabinoxylan [10], consisting of a xylose backbone substituted with different side chains such as arabinose, glucuronic acid, or acetic acid [11,12,13]. Therefore, several enzymes are necessary to remove the sterically hindering side chains (arabinofuranosidase, glucuronidase, acetyl xylan esterase), cleaving the bonds of the backbone (endoxylanase) and further degrading the released oligomers (β-xylosidase) [14,15,16,17].

Starch is a glucose polymer and appears as unbranched, helical amylose chains or as branched amylopectin, once again deeming several enzymatic activities necessary for its decomposition. One group of starch-degrading enzymes is α-amylases, which are able to hydrolyze random glycosidic bonds within the polymeric and oligomeric glucose chains [18]. The produced dimer maltose is consequently degraded by the α-glucosidase or maltase [19]. Another way of action is demonstrated by the γ-amylases, which are releasing single glucose molecules from the non-reducing chain ends by successive cleavage of the final glycosidic bonds [18]. Nowadays, amylases are produced in large amounts, e.g., with the filamentous fungus *A. niger* and applied for various industrial purposes [20,21,22,23].

The third polymer in the wheat bran particles is cellulose. It consists of β-1,4-linked glucose chains which are connected via hydrogen bonds to form crystalline fibers, the microfibrils. A variety of enzymes is necessary to break down cellulose, with the main types being endo-glucanases, exo-glucanases or cellobiohydrolases, and β-glucosidases [24,25]. Cellulose degradation begins with the endo-glucanases cleaving glycosidic bonds in amorphous regions of the polymer. Subsequently, cellobiose molecules are released from the non-reducing ends of the glucan chains by the exo-glucanases [25,26,27]. The final conversion of cellobiose to glucose molecules is catalyzed by β-glucosidase [25].

The complex degradation of agricultural residues like wheat bran is opposed by the fact that most microorganisms are specialized in the production of only a few hydrolytic enzymes. As the production of all necessary enzymes in several separate processes would be extremely labor- and cost-intensive [28], the co-cultivation of the respective microorganisms poses a promising alternative approach to produce the desired enzyme mixture [29,30]. In an earlier study, we reported the establishment of such a co-culture consisting of the two filamentous fungi *Aspergillus niger* and *Trichoderma reesei* for the hydrolysis of wheat bran [31]. The ascomycete *A. niger* has been one of the most researched and applied microorganisms in biotechnology and is well-known for its capability to produce several enzymes as well as organic acids [32,33,34]. In the case of the degradation of lignocellulosic materials like wheat bran, especially the xylanases and amylases produced by *A. niger* are of great interest. *A. niger* NRRL 2270 (ATCC 11414), which was used in the presented work, stems from a citric acid-producing strain [35], but nevertheless, a high potential regarding the enzymatic degradation of plant material has been observed and reported [31,36]. This strain was partnered with *T. reesei*, which is known for its high capability for cellulase formation and secretion [37,38]. The specific strain *T. reesei* RUT-C30 was isolated after a series of mutagenesis steps with subsequent selection of cellulase producers [39]. This led to catabolite de-repression in the organism, resulting in high secretion of cellulolytic enzymes into the medium, which made the strain highly interesting to many studies throughout the years [40,41,42,43,44,45]. However, the randomly induced mutations also resulted in a reduction in amylase and β-glucosidase formation [39,46,47]. This deficiency can be compensated by other microorganisms, including *A. niger.* Overall, the production of xylanases, amylases, and β-glucosidase by *A. niger* NRRL 2270, combined with the cellulase hypersecretion of *T. reesei* RUT-C30, results in a complementary and adapted enzyme mixture to hydrolyze wheat bran [30,31,48,49,50,51,52].

An additional challenge in converting agricultural residues into sugar monomers usable by other microorganisms is posed by the necessity of two separate processes for enzyme production and hydrolysis [30]. This results in a number of process steps, including the separation and stabilization of the enzyme solution, as well as several sterilization and cleaning steps or the availability of multiple bioreactor systems, both leading to a high demand in labor and equipment. These steps can, however, be eliminated by the integration of enzyme production and hydrolysis into a one-pot process, lowering the total process time and costs. In order to avoid consumption of the released sugars during the hydrolysis by the enzyme-producing fungi, the reaction conditions can be changed to unfavorable conditions for growth before the addition of the biomass for hydrolysis [53,54,55].

The hydrolysate produced during the process, mainly containing the sugars D-glucose and xylose, can subsequently serve as the substrate for other microbial conversion processes. Various studies have demonstrated the production of second-generation bioethanol from wheat bran hydrolysates via ABE (acetone–butanol–ethanol) fermentation with, e.g., *Clostridium* sp. or yeasts [56,57,58,59]. Aside from ethanol, other chemicals such as lactic acid can be produced as well by applying several microorganisms like *Lactobacillus* spp. [60,61,62], and a variety of further microbial products appear feasible with the utilization of the released sugars. Under certain circumstances, e.g., safety regulations regarding the microorganisms used, the residual solid biomass is applicable as animal feed in order to achieve a real waste-free process. As the substrate, in this case wheat bran, would also have been fed to animals, this way of processing means an improved valorization of the agricultural residues.

This study aimed for a one-pot process for the valorization of wheat bran with a co-culture of *A. niger* and *T. reesei*. Initially, the process was established in stirred tank bioreactors on a 25 L scale, demonstrating the simple integration of enzyme production and hydrolysis. At this stage, the remaining solid biomass was analyzed for its nutritional properties and was found to be a high-quality animal feed. Consequently, the process was successfully scaled up to the 1000 L scale. This transfer to a geometrically non-similar stirred tank bioreactor resulted in lower yields than on the lab scale but showed the feasibility of the integrated process on a larger scale. Additionally, the issue of biomass separation after the integrated process was tackled to produce, on the one hand, a sugar-rich hydrolysate that may be useful as a substrate for various biotechnological processes and, on the other hand, a solid animal feed with improved properties.

## 2. Materials and Methods

### 2.1. Strains and Media

*Aspergillus niger* (*A. niger*) NRRL 2270 was obtained from the NRRL strain collection (Peoria, Illinois, USA), and *Trichoderma reesei* (*T. reesei*) RUT-C30 was kindly provided by the Werner Siemens Chair of Synthetic Biotechnology, Technical University of Munich, Garching, Germany. Spores of *A. niger* were grown on 39 g L^−1^ potato extract dextrose agar (Carl Roth GmbH + Co. KG, Karlsruhe, Germany) supplemented with 10 g L^−1^ yeast extract and 1 mL L^−1^ trace element solution for 5 d. *T. reesei* spores were cultivated for 7 d on potato extract dextrose agar without supplements. Spores were harvested by washing them off the agar plates with sterile 0.89% NaCl solution with 0.05% Tween 80. Spore suspensions were subsequently filtered through sterile cotton wool, washed, and mixed with 30% glycerol for storage at −80 °C.

The preculture medium contained 10 g L^−1^ yeast extract and 10 mM of xylose, maltose, and lactose. The other components of the medium were (L^−1^) 23.1 g (NH_4_)_2_SO_4_, 1.5 g KH_2_PO_4_, 0.5 g KCl, 0.5 g MgSO_4_ ∙ 7 H_2_O, 1 mL L^−1^ trace element solution, and 0.1% (*v*/*v*) PPG P2000 as antifoaming agent. The trace element solution consisted of (L^−1^) 10 g EDTA, 4.4 g ZnSO_4_ ∙ 7 H_2_O, 1.01 g MnCl_2_ ∙ 4 H_2_O, 0.32 g CoCl_2_ ∙ 6 H_2_O, 0.32 g CuSO_4_ ∙ 5 H_2_O, 0.22 g (NH_4_)_6_Mo_7_O_24_ ∙ 4 H_2_O, 1.47 g CaCl_2_ ∙ 2 H_2_O, and 1 g FeSO_4_ ∙ 7 H_2_O [63].

All cultivations described for enzyme production were conducted in minimal medium with 30 g L^−1^ milled and pre-dried wheat bran (kindly provided by Bayerischer Müllerbund, Munich, Germany) as carbon source. The other components were identical to those of the preculture medium.

### 2.2. Preculture Preparation in Shaking Flasks

Precultures for the 25 L cultivations, as well as for the bioreactor precultures for the 1000 L process, were cultivated in 1 L shaking flasks without baffles containing 200 mL preculture medium. *A. niger* precultures were inoculated using 10^9^ spores L^−1^ and were incubated at 30 °C and 180 min^−1^ shaking rate for 48 h. *T. reesei* precultures were started by inoculation with 0.2 × 10^9^ spores L^−1^ and were incubated for 72 h at the same conditions. The precultures were subsequently mixed in a sterile glass bottle at the desired biomass ratio and used for inoculation. Achievable biomass concentrations in the shake flasks were determined in repeated cultivations beforehand to enable the calculation of the necessary preculture volumes.

### 2.3. Integrated Enzyme Production and Hydrolysis Process on the 25 L Scale

Batch cultivation processes on a 25 L scale were carried out in a 42 L stirred tank bioreactor (Techfors, Infors HT, Bottmingen, Switzerland) with four baffles and a stirrer configuration of (from top to bottom) one downwards-pumping inclined blade stirrer, one upwards-pumping inclined blade stirrer, and one 6-blade Rushton turbine for sufficient homogenization. Sterile air was provided via a ring sparger at the vessel bottom; 750 g pre-dried wheat bran was suspended in 20 L desalted water before in situ sterilization (120 °C, 20 min). Other components of the minimal medium were added aseptically afterwards via peristaltic pumps. The enzyme production process was started by inoculation with the mixed preculture of *A. niger* NRRL 2270 and *T. reesei* RUT-C30 with an initial overall biomass concentration of 1.2 g L^−1^ (1.0 g L^−1^ *A. niger*, 0.2 g L^−1^ *T. reesei*) and was conducted at 30 °C. During cultivation, pH 4.5 was kept constant by a controller using 1 M H_2_SO_4_ and 3 M KOH as titration agents. In order to keep the dissolved oxygen concentration above 30% air saturation, the aeration rate was increased stepwise from 5 L min^−1^ to 43 L min^−1^ (0.2–1.72 vvm). In addition, the pressure was increased from 1 bar to 2.2 bar at a process time of 17 h, followed by dynamic control of the agitation rate between 330 and 600 min^−1^ (stirrer tip speed 1.9–3.5 m s^−1^) with an asymmetrical proportional controller, in which the reduction of the stirring rate was steeper in order to avoid extensive shear stress. Process control and data monitoring and recording were realized via the IRIS Control Software (V5.3, Infors HT, Bottmingen, Switzerland).

The end of the enzyme production was indicated by a rapid increase in DO due to the depletion of nutrients. At this point, the temperature was increased to a hydrolysis temperature of 50 °C, which was shown previously to be the optimal temperature for the present enzyme mixture [31]. Simultaneously, the agitation rate was set to 550 min^−1^, the pressure was reduced to atmospheric pressure, and the aeration rate was lowered to the minimum value of 5 L min^−1^ to prevent blockage of the sparger by biomass particles. After 1 h, the vessel was opened at the reactor lid and 2.5 kg pre-dried, unsterile wheat bran was added via a funnel, and the reactor was closed again. Meanwhile, pH control was paused until the broth was completely homogenized.

Samples were taken regularly during both process phases through a steam-sterilizable sample valve at the bottom of the reactor. An additional sample of 5 L was taken at the end of the one-pot process, the remaining biomass and the hydrolysate supernatant were separated by centrifugation (3260× *g*, 30 min, 4 °C) and decanting, and the resulting solid biomass was stored at −20 °C until delivery to the Bavarian State Institution for Agriculture (Bayerische Landesanstalt für Landwirtschaft LfL, Grub/Poing, Germany) for the standardized lab analysis regarding feed-relevant components.

The wheat bran composition was analyzed by the Werner Siemens Chair of Synthetic Biotechnology, TUM, Garching, Germany.

### 2.4. Preculture Preparation in Stirred Tank Bioreactors for Cultivation on the 1000 L Scale

Two separate stirred tank bioreactors were used to simultaneously prepare the precultures of the two strains for the 1000 L process.

The preculture of *A. niger* NRRL 2270 was cultivated in a stirred tank bioreactor with a maximum working volume of 50 L (LP75L, Bioengineering, Wald, Switzerland). The reactor had four baffles and two 6-blade Rushton turbines, a ring sparger, and sensors for relevant process parameters and was controlled via BioSCADALab software (Bioengineering, Wald, Switzerland). The reactor was filled with 40 L desalted water and 500 g yeast extract before in situ sterilization (120 °C, 20 min); all other components of the preculture medium were autoclaved separately and added aseptically via pumps. Inoculation was carried out with 1.2 L shaking flask preculture being pumped into the reactor. The temperature was held at 30 °C, and a constant pH 4.5 was maintained by automated titration with 1 M H_2_SO_4_ and 3 M KOH. The agitation rate was kept constant at 200 min^−1^, and oxygen was provided by aeration with 25–67 L min^−1^ air and 0.7 bar overpressure. The batch process duration was 22 h before the culture was used as inoculum for the process on a 1000 L scale.

*T. reesei* preculture was prepared in a stirred tank bioreactor with 25 L working volume (Techfors, Infors HT, Bottmingen, Switzerland); 250 g yeast extract was suspended in 19 L desalted water inside the vessel prior to in situ sterilization, and the preculture medium was then complemented by sterile addition of other medium components. Then, 0.8 L of shaking flask preculture was used as inoculum. The batch cultivation was conducted at 30 °C and a constant pH 4.5, automatically controlled by the addition of 1 M H_2_SO_4_ and 3 M KOH. The DO was held above 30% air saturation by stepwise increasing of the gassing rate (5–40 L min^−1^) and dynamically controlling the stirrer speed (330–475 min^−1^); 0.6 bar overpressure was applied additionally after 13 h process time. The batch cultivation lasted for 34 h, and the broth was subsequently transferred into sterile containers for inoculation of the 1000 L process.

### 2.5. Integrated Enzyme Production and Hydrolysis Process on the 1000 L Scale

The integrated enzyme production and hydrolysis process on a pilot scale was conducted in a stirred tank bioreactor with a maximum working volume of 1000 L (LP1500, Bioengineering, Wald, Switzerland). The reactor was equipped with four equidistant baffles and three Rushton turbines for homogenization and a ring sparger at the bottom for aeration. Prior to in situ sterilization, 30 kg pre-dried wheat bran was suspended in 750 L desalted water. Other media components (in total 175 L) were sterilized in separate medium tanks and were transferred to the reactor after cooling to 30 °C. The *A. niger* preculture (50 L) was transferred into the reactor via a direct transfer tube and overpressure inside the preculture reactor. Simultaneously, 25 L of *T. reesei* preculture was added with a peristaltic pump from the transport containers, resulting in an initial biomass concentration of 1.2 g L^−1^ and a ratio of 5:1 in favor of *A. niger*. Temperature was set to 30 °C during enzyme production, an overpressure of 0.7 bar was applied, and the pH was controlled at 4.5 by automated titration with 1 M H_2_SO_4_ or 3 M KOH. A DO control cascade controlling the aeration from 400–2000 L min^−1^ (0.4–2.0 vvm) and stirring rate from 70–200 min^−1^ (corresponding to 1.3–3.8 m s^−1^ stirrer tip speed) served for sufficient oxygen supply (DO > 30% air saturation). The complete process was controlled and recorded by the control software BioSCADALab (Bioengineering, Wald, Switzerland).

A strong increase in DO indicated the depletion of nutrients and thus marked the end point of the enzyme production. Hence, the temperature was increased to 50 °C, the stirring rate was set to 150 min^−1^, the overpressure was released, and the aeration was switched off. When 50 °C was reached, the reactor was opened at the lid and 100 kg of dried wheat bran was added. The pH control was paused until complete homogenization to prevent excess titration during homogenization.

Samples were withdrawn through a sterilizable sampling valve at the bottom of the vessel.

The process was terminated by finishing agitation and control of pH and temperature. After 1 h of sedimentation of the remaining solids, the vessel lid was lifted, and the supernatant was decanted using a membrane pump (SartoJet, Sartorius AG, Göttingen, Germany). A pre-filtering device (pore size 1.2 mm, quadratic) mounted at the end of the hose dived into the liquid phase of the reactor was used to retain larger biomass and wheat bran particles. The harvested hydrolysate was pumped through a plate separator (CSA 8-06-476, GEA Westfalia Separator Group GmbH, Oelde, Germany) to further remove smaller particles and then collected in a medium tank. The separator was operated at a flow rate of 300 L h^−1^, and the solids were discharged for 200–250 ms every 410–450 s until no more solid discharge was observed.

### 2.6. Analytical Methods

#### 2.6.1. Sampling and Sample Treatment

Samples were withdrawn from the bioreactors at various time points using the sampling outlets of the respective bioreactors, aliquoted into 2 mL reaction tubes, and stored on ice. Fungal biomass and residual wheat bran particles were removed by centrifugation at 21,000× *g* for 20 min at 4 °C and subsequent filtration (0.2 µm). The filtrated supernatants were then stored at −20 °C until further analysis.

#### 2.6.2. Hydrolytic Enzyme Activities

Total xylanase, amylase, and cellulase activities were determined by colorimetric assay using 4-hydroxybenzhydrazide (pHBAH), following a protocol previously described [31] measuring the sugar release from xylan, starch, and carboxymethyl cellulose (CMC), respectively. Activities of each sample were determined in triplicate and results were depicted as means with respective standard deviations.

#### 2.6.3. Quantification of Sugars and Organic Acids

Concentrations of relevant monosaccharides, disaccharides, and organic acids in the supernatant samples were determined by the high-performance liquid chromatography method reported by Mittermeier et al. [31]. 

#### 2.6.4. Calculation of Carbon Dioxide Evolution Rate (CER)

The carbon dioxide evolution rate as measure for metabolic activity was calculated as follows:

(1) $$CER=-\frac{{\dot{V}}_{g,in}}{V_{L}V_{m}}(x_{{CO}_{2},in}-\frac{1-x_{{CO}_{2},in}-x_{O_{2},in}}{1-x_{{CO}_{2},out}-x_{O_{2},out}}x_{{CO}_{2},out})$$

with the gas flow rate into the reactor ${\dot{V}}_{g,in}$, the reaction volume $V_{L}$, the molar volume of an ideal gas (22.4 L mol^−1^) $V_{m}$, and the concentrations of carbon dioxide and oxygen coming into (in) and out (out) of the reactor $x_{{CO}_{2},in}$, $x_{O_{2},in}$, $x_{{CO}_{2},out}$, and $x_{O_{2},out}$, respectively [64]. 

## 3. Results

### 3.1. Integrated Enzyme Production and Hydrolysis of Wheat Bran on a 25 L Scale

**Figure 1:**
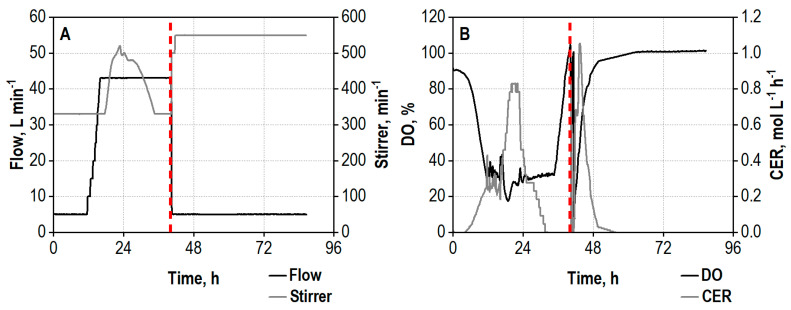
(**A**) Air flow rate (Flow), L min^−1^, and agitation rate (Stirrer), min^−1^; (**B**) dissolved oxygen concentration (DO), %, and carbon dioxide evolution rate (CER), mol L^−1^ h^−1^, during co-cultivation of *A. niger* NRRL 2270 and *T. reesei* RUT-C30 on the 25 L scale using 30 g L^−1^ wheat bran, followed by integrated hydrolysis at 50 °C. The process was started by inoculation from shake flasks with an initial biomass concentration of 1.2 g L^−1^ and a ratio of *A. niger* to *T. reesei* of 5:1. The dashed line marks the increase in temperature to 50 °C and addition of 100 g L^−1^ wheat bran for the integrated hydrolysis process.

The integrated process of enzyme production and hydrolysis based on wheat bran was first studied in a stirred tank reactor on a 25 L scale. The growth of the fungi began shortly after inoculation without a lag phase, observable by a decreasing oxygen saturation of the medium and commencing carbon dioxide evolution (Figure 1B). The DO control cascade subsequently managed to avoid oxygen limitations of the cells by stepwise increasing of the aeration rate to the maximum of 43 L min^−1^ between a process time of 11.5–16 h. Additionally, an overpressure of 1.2 bar was applied after 17 h cultivation time, and the dynamic stirrer control cascade regulated the agitation rate to keep the DO above the critical threshold of 30% air saturation (Figure 1A). The maximum metabolic activity in the process could be observed between 20 and 24 h by the peak in the CER (Figure 1B). The rapid increase in DO after 35 h points at the depletion of the accessible carbon sources and thus the end of the enzyme production phase.

**Figure 2:**
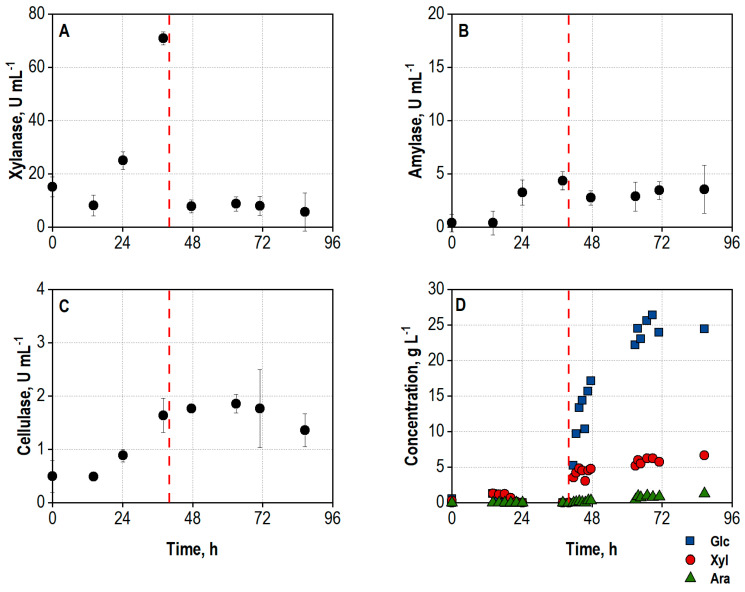
(**A**) Total xylanase activity, U mL^−1^; (**B**) total amylase activity, U mL^−1^; (**C**) total cellulase activity, U mL^−1^; (**D**) concentrations of D-glucose (Glc), D-xylose (Xyl), and L-arabinose (Ara), g L^−1^, in the supernatant during co-cultivation of *A. niger* NRRL 2270 and *T. reesei* RUT-C30 in a stirred tank bioreactor on a 25 L scale using 30 g L^−1^ wheat bran, followed by integrated hydrolysis at 50 °C. The process was started by inoculation from shake flasks with an initial biomass concentration of 1.2 g L^−1^ and a ratio of *A. niger* to *T. reesei* of 5:1. The dashed line marks the increase in temperature to 50 °C and addition of 100 g L^−1^ wheat bran for the integrated hydrolysis process.

Smaller hydrolytic activities were already present at inoculation due to enzymes produced during preculture preparation (Figure 2A–C). All three enzyme activities necessary to efficiently degrade the polysaccharides in wheat bran (xylanase, amylase, and cellulase) were produced simultaneously within the enzyme production phase, with xylanase reaching the highest activity measured. This fact also indicates growth and secretion activity of both *A. niger* and *T. reesei* within the co-culture.

The enzyme production was terminated after 40 h by increasing the temperature to 50 °C, releasing the overpressure and reducing the aeration rate to a minimum rate of 5 L min^−1^ to prevent a blockage of the gas openings of the sparger. This resulted in a short drop of the DO, followed by an increase due to the inactivation of the organisms. The second peak of the CER can be explained by the reduced air flow, resulting in higher CO_2_ concentrations in the exhaust gas and thus a higher CER. The DO subsequently reaches 100%, meaning that no more oxygen is consumed by the fungi, and the CO_2_ production stops as well. Unsterile wheat bran was added for the hydrolysis 1 h after increasing the temperature to 50 °C, and the agitation rate was set to 550 min^−1^ to ensure homogenization. The addition of wheat bran led to a strong reduction of xylanase activity in the supernatant samples, most probably caused by adsorption of the enzymes to the substrate particles [65]. The release and accumulation of sugars starts almost immediately after the inactivation of the fungi and addition of wheat bran (Figure 2D) and already summed up to 22.2 g L^−1^ after 6 h of hydrolysis. An overall sugar concentration of 32.3 g L^−1^, consisting of 24.4 g L^−1^ D-glucose, 6.7 g L^−1^ D-xylose, and 1.2 g L^−1^ L-arabinose, was measured at the end of the integrated process after 86.6 h, corresponding to approx. 59% of the theoretical yield.

An additional sample with a volume of 5 L was taken at the end of the process. The residual solid fraction was separated from the resulting hydrolysate and analyzed regarding its nutritional potential for animals. The composition of raw wheat bran and the treated biomass is listed in Table 1. 

Overall, the fermentation residues demonstrated improved properties for animal nutrition compared to untreated wheat bran, mainly due to increased concentrations of valuable nutrients. The protein content was 48% higher than in the raw material, which is caused by the secretion of hydrolytic enzymes by the fungal strains. Lipids were released from the wheat bran particles after degradation of the polysaccharide matrix, increasing the fat content by 85%. Additionally, the content of available sugars was almost three times as high (+190%). This resulted in an increased energy density of the biomass, which rose, e.g., for ruminants from 9.69 MJ kg^−1^ to 12.52 MJ kg^−1^. An additional beneficial effect is the reduction of phosphate by 38%, which, on the one hand, reduces the phosphate deposition into the environment and thus helps to prevent eutrophication of soils and water and, on the other hand, enables better animal nutrition in accordance with environmental laws.

**Figure 3:**
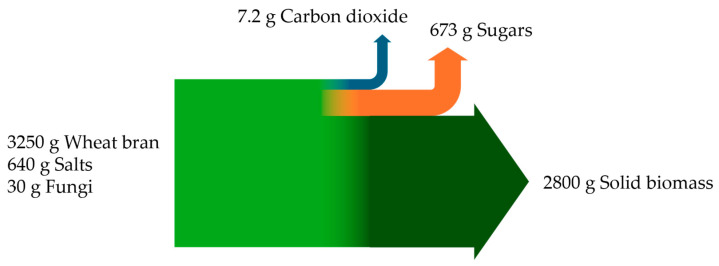
Flow diagram displaying carbon flux in and out of the integrated enzyme production and hydrolysis process on a 25 L scale.

A carbon mass balance was estimated based on the mass streams of carbon compounds getting in and out of the process during the process. The main input of carbon was wheat bran with a mass of 3250 g, divided into 750 g for the enzyme production and 2500 g for the hydrolysis. Other relevant inputs in the process were 640 g salts partly assimilated into the biomass and 30 g of fungal cells as inoculum. A total of 7.2 g carbon dioxide was formed during the growth and enzyme production of the microorganisms (quantified by exhaust gas analysis), and an overall sugar mass of 673 g in the hydrolysate was determined at the end of the hydrolysis based on HPLC data. The larger sample (5 L), taken at the end of the process for solid biomass analysis, contained ≈3 kg wet biomass after separation with a dry biomass share of 18.7% (*w*/*w*, data from Bavarian State Institution for Agriculture, LfL). This accounts for approx. 2800 g dry biomass when projected on the total process volume, closing the balance (Figure 3).

### 3.2. Integrated Enzyme Production and Hydrolysis of Wheat Bran on a 1000 L Scale

**Figure 4:**
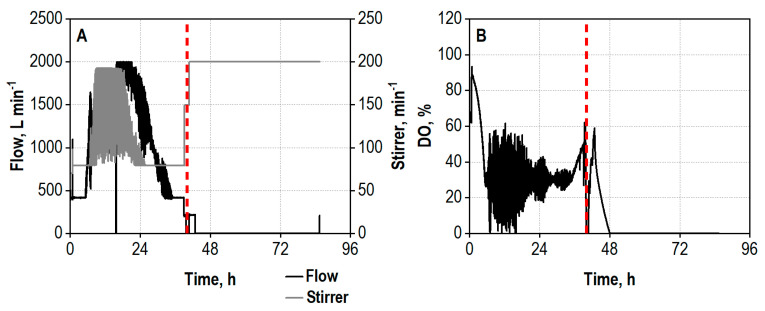
(**A**) Air flow rate (Flow), L min^−1^, and agitation rate (Stirrer), min^−1^; (**B**) dissolved oxygen concentration (DO), %, during co-cultivation of *A. niger* NRRL 2270 and *T. reesei* RUT-C30 in a strirred tank bioreactor on a 1000 L scale using 30 g L^−1^ wheat bran, followed by integrated hydrolysis at 50 °C. The process was started by inoculation from the preculture reactor (*A. niger*) or from the transport containers (*T. reesei*) with an initial biomass concentration of 1.2 g L^−1^ and a ratio of *A. niger* to *T. reesei* of 5:1. The dashed line marks the increase in the temperature to 50 °C and addition of 100 g L^−1^ wheat bran for the integrated hydrolysis process.

The demonstration of the technical feasibility of the integrated process was conducted on a 1000 L scale. The transfer of the co-cultivation of *A. niger* and *T. reesei* was based on constant volumetric aeration rate. The initial stirrer tip speed was, however, decreased compared to the 25 L scale to avoid excessive shear forces from the Rushton turbines in the reactor. The growth of the fungi commenced without a noteworthy lag phase, observable by the declining oxygen saturation in the medium (Figure 4B). High noise occurred regarding the aeration and agitation in course of the DO control due to the increased viscosity of the broth and technical issues with the exhaust gas filter (Figure 4A), nevertheless longer periods of oxygen limitation for the cells could be avoided (Figure 4B). The beginning rise of the DO concentration after 36 h indicated the end of the enzyme production phase, and the hydrolysis phase was started by setting the temperature to 50 °C, increasing the stirring rate as well as terminating the aeration.

**Figure 5:**
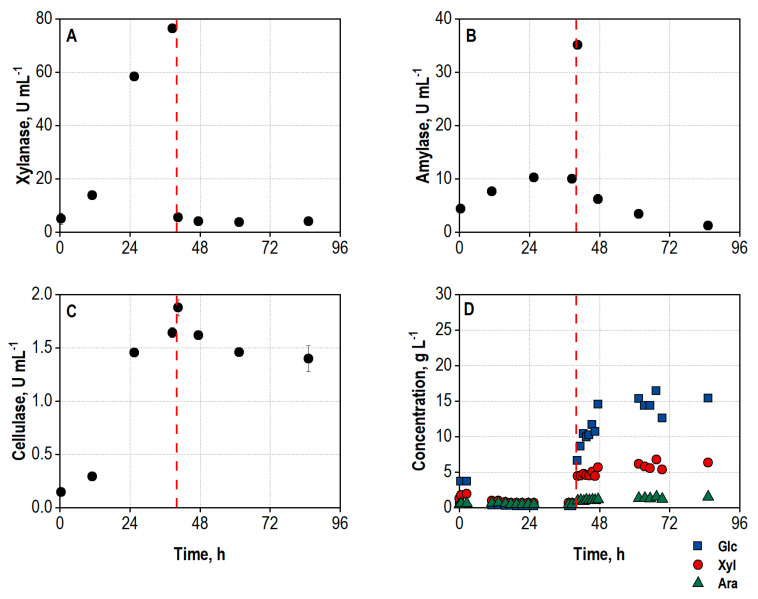
(**A**) Total xylanase activity, U mL^−1^; (**B**) total amylase activity, U mL^−1^; (**C**) total cellulase activity, U mL^−1^; (**D**) concentrations of D-glucose (Glc), D-xylose (Xyl), and L-arabinose (Ara), g L^−1^, in the supernatant during co-cultivation of *A. niger* NRRL 2270 and *T. reesei* RUT-C30 in a stirred tank bioreactor on a 1000 L scale using 30 g L^−1^ wheat bran, followed by integrated hydrolysis at 50 °C. The process was started by inoculation from the preculture reactor (*A. niger*) or from the transport container (*T. reesei*) with an initial biomass concentration of 1.2 g L^−1^ and a ratio of *A. niger* to *T. reesei* of 5:1. The dashed line marks the increase in temperature to 50 °C and addition of 100 g L^−1^ wheat bran for the integrated hydrolysis process.

The enzyme secretion occurred faster than in the smaller process due to the missing lag phase, but, especially, the amylase activity appeared to reach a saturation after 25 h (Figure 5A–C). At the end of the enzyme production process, the amylase activity was 132% higher than on the 25 L scale, whereas the xylanase and cellulase activities remained at the same level (xylanase + 7%, cellulase ± 0%).

The addition of 100 g L^−1^ wheat bran for hydrolysis was conducted after a process time of 39 h. The xylanase activity in the supernatant dropped immediately due to adsorption of the enzymes to the wheat bran particles. Amylases already present in the wheat bran and active due to no sterilization of the biomass before addition simultaneously caused a sudden peak in amylase activity. The sugar release also started instantly, and a total sugar concentration of 15.8 g L^−1^ was already measured after 6 h hydrolysis time (Figure 5D). After 85 h total process time (46 h hydrolysis), the overall sugar concentration summed up to 23.3 g L^−1^. The hydrolysate contained 15.4 g L^−1^ D-glucose, 6.4 g L^−1^ D-xylose, and 1.5 g L^−1^ L-arabinose, corresponding to 42% of the theoretical yield.

**Figure 6:**
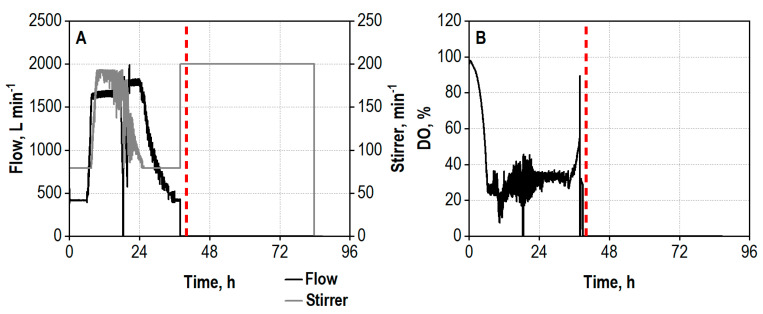
(**A**) Air flow rate (Flow), L min^−1^, and agitation rate (Stirrer), min^−1^; (**B**) dissolved oxygen concentration (DO), %, during the repeated co-cultivation of *A. niger* NRRL 2270 and *T. reesei* RUT-C30 in a stirred tank bioreactor on a 1000 L scale using 30 g L^−1^ wheat bran, followed by integrated hydrolysis at 50 °C. The process was started by inoculation from the preculture reactor (*A. niger*) or from the transport container (*T. reesei*) with an initial biomass concentration of 1.2 g L^−1^ and a ratio of *A. niger* to *T. reesei* of 5:1. The dashed line marks the increase in temperature to 50 °C and addition of 100 g L^−1^ wheat bran for the integrated hydrolysis process.

The process on a 1000 L scale was repeated to prove its reproducibility. Again, growth of the fungi began without a delay directly after inoculation (Figure 6B). The DO control occurred less noisily, resulting in shorter periods of DO concentrations below 30% air saturation. Additionally, the agitation rate was mostly lower than in the first process, leading to a lower shear force input (Figure 6A). The growth and enzyme production ended after 35 h, indicated by a rapid DO increase.

**Figure 7:**
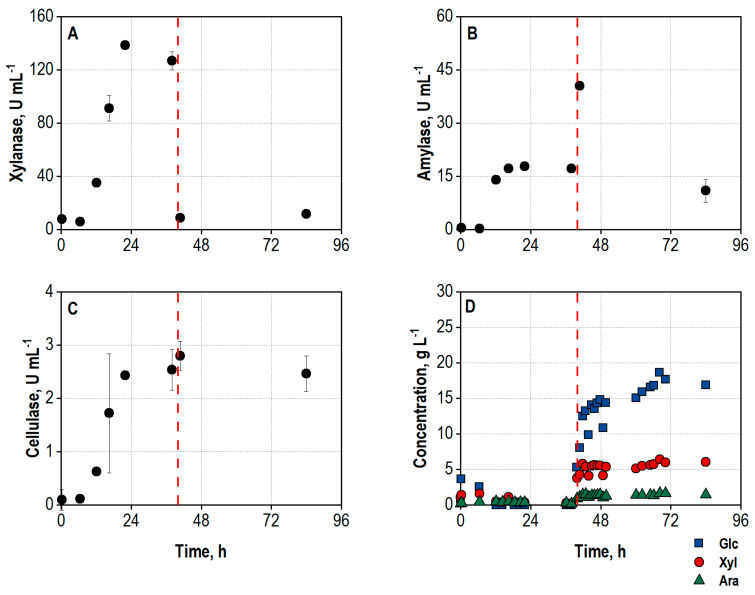
(**A**) Total xylanase activity, U mL^−1^; (**B**) total amylase activity, U mL^−1^; (**C**) total cellulase activity, U mL^−1^; (**D**) concentrations of D-glucose (Glc), D-xylose (Xyl), and L-arabinose (Ara), g L^−1^, in the supernatant during the repeated co-cultivation of *A. niger* NRRL 2270 and *T. reesei* RUT-C30 in a stirred tank bioreactor on a 1000 L scale using 30 g L^−1^ wheat bran, followed by integrated hydrolysis at 50 °C. The process was started by inoculation from the preculture reactor (*A. niger*) or from the transport container (*T. reesei*) with an initial biomass concentration of 1.2 g L^−1^ and a ratio of *A. niger* to *T. reesei* of 5:1. The dashed line marks the increase in temperature to 50 °C and addition of 100 g L^−1^ wheat bran for the integrated hydrolysis process.

Surprisingly, the formation of the hydrolytic enzyme activities appeared accelerated compared to the first 1000 L process (Figure 7A–C) despite a similar course of the process. Also, the determined activities at the end of the enzyme production phase were significantly higher than in the first process, and increases of 66%, 73%, and 56% were determined for xylanase, amylase, and cellulase, respectively. Possible reasons for these drastic changes include the differences in shear force input and oxygen supply, or the application of a different lot of wheat bran.

The enhanced enzyme activities apparently resulted in a more rapid release of sugars from wheat bran added for hydrolysis after a process time of 39.8 h. Already after 6 h of hydrolysis, a total concentration of 20.4 g L^−1^ sugar monomers had been accumulated. Nevertheless, the final sugar concentration in the hydrolysate reached 24.4 g L^−1^ (44% yield) and thus was only slightly higher than in the first process (+5%). The hydrolysate was composed of 16.9 g L^−1^ D-glucose, 6.1 g L^−1^ D-xylose, and 1.4 g L^−1^ L-arabinose. 

Although the enzyme activities were quite different, the comparable sugar concentration as the final outcome of the integrated process showed reproducibility within the estimation error. The reduced final sugar concentrations compared to the integrated process in a stirred tank bioreactor on a 25 L scale could be caused by differences between the scales (e.g., stirrer configurations, no geometric similarity, variations in process control).

A combination of sedimentation, decanting and centrifugation with a plate separator enabled the separation of hydrolysate and residual solids and resulted in a total volume of 580 L hydrolysate. An immediate stabilization of the hydrolysate by thermal sterilization was necessary due to the unsterile harvest procedure. A direct separation of the solids with the plate separator was not feasible due to the high solids content, which instantly blocked the discharge container.

## 4. Discussion

In this study, we demonstrated an integrated process for the valorization of wheat bran by enzymatic saccharification, resulting in sugars and improved animal feed, up to the technical 1000 L scale. Scalability to even larger scales appears feasible due to the simple character of the process, as there are no specific requirements regarding the bioreactor, and an easily available low-price substrate is used. The process integration is enabled by the change in temperature from 30 °C for enzyme production to 50 °C for the hydrolysis. On the one hand, this increased temperature inactivates the fungal cells in the broth [66]; on the other hand, it creates optimal process conditions for the hydrolytic enzyme mixture produced [31]. Those two factors lead to the accumulation of released sugars in the hydrolysate in concentrations similar to or even higher than in comparable studies, without harsh pretreatments such as steam explosion or extrusion [67,68]. As this hydrolysate mainly contains the easily convertible sugars D-glucose and D-xylose, it can serve as substrate for biotransformation processes to a broad variety of products. For instance, several studies reported the conversion of wheat bran hydrolysate to lactic acid using different microorganisms [60,61,62]. Another relevant approach in recent research is posed by the production of biofuels or solvents from the released sugars via ABE (acetone–butanol–ethanol) fermentation, e.g., with *Saccharomyces cerevisiae* or *Clostridium* spp. [56,57,58,59,69]. Since many microorganisms are known to convert D-glucose and D-xylose, the fermentative production of bulk and fine chemicals from wheat bran hydrolysate still can be expanded to further utilize this abundant resource. In addition, L-arabinose can also be converted mixotrophically to meso-2,3-butanediol, a monomer of polyurethanes, using anaerobic syngas fermentation [70].

As the hydrolysate has relatively low sugar concentrations, it may be necessary to evaporate excess water to achieve higher product concentrations in batch fermentation processes. The concentrated hydrolysate is, in addition, enriched with all other soluble nutrients (amino acids, peptides, phosphate, ammonia, and others) and may thus be applied as a fermentation medium with reduced need to supplement salts or other nutrients, offering lower costs for subsequent production processes and thus improving their economic feasibility. However, process challenges may be posed by the aforementioned issues in transferring fungal processes to different reactor systems or by the separation of the unstable hydrolysate and the residual biomass in a short time with sufficient efficiency. 

The other product originating from the integrated process is the solid biomass containing residual fermented wheat bran and fungal cells. The composition analysis presented in this work suggest that this protein-rich biomass can be applied as a high-quality animal feed. This is enabled by the application of well-studied wild-type strains of fungi that are already described and certified as safe for the use in food and feed [71,72]. As reviewed by Ghorai et al. in 2009, enzymes stemming from *A. niger* or *T. reesei* have been used in food and feed processing in many processes and for a long time [73]. Other studies have also described the positive effects that the use of fungi has on animal feed regarding the nutrient availability, protein content, and other functional components [74,75].

## 5. Conclusions

The integrated process presented utilizes the abundant raw material wheat bran and converts it into two products, sugars and improved animal feed. The release and separation of the sugar-containing hydrolysate opens the possibility to turn a share of the wheat bran into products of higher value, such as platform chemicals or biofuels, by fermentation of the sugar-rich hydrolysate. The residual fermented biomass is applicable as animal feed with even improved properties regarding the nutritional value, as well as its environmental impact. The scale-up to the 1000 L scale demonstrated the feasibility of the integrated one-pot process in larger volumes and could provide the basis for its realization in an industrial context.

## Figures and Tables

**Table 1:** Composition of raw wheat bran and fermentation residues from the integrated enzyme and hydrolysis process on a 25 L scale.

		Raw Wheat Bran	Fermentation Residues
Protein	g kg^−1^	156	231
Sugar	g kg^−1^	29	84
Fat	g kg^−1^	33	61
Phosphate (P_2_O_5_)	g kg^−1^	33.9	20.9
Energy content (ruminants)	MJ kg^−1^	9.69	12.52

## Data Availability

The original contributions presented in the study are included in the article; further inquiries can be directed to the corresponding authors.
